# MSA-CNN: A Multi-Scale Attention Convolutional Neural Network for fNIRS-Based Emotion Recognition

**DOI:** 10.3390/bios16080434

**Published:** 2026-08-09

**Authors:** Deping Huang, Xiu Zhang, Ye Li, Jingfu Wu, Youzhi Yue

**Affiliations:** 1 College of Electronic and Communication Engineering, Tianjin Normal University, Tianjin 300387, China; hh256212@126.com (D.H.); ly20010225@outlook.com (Y.L.); haoshuaishuaiwo@163.com (J.W.); 18554617128@163.com (Y.Y.); 2 Tianjin Key Laboratory of Wireless Mobile Communications and Power Transmission, Tianjin Normal University, Tianjin 300387, China

**Keywords:** functional near-infrared spectroscopy, emotion recognition, multi-scale features, attention mechanism, subject-independent evaluation

## Abstract

Functional near-infrared spectroscopy (fNIRS) has attracted increasing attention in affective brain–computer interface research due to its non-invasive nature, portability, and robustness to motion artifacts. However, substantial inter-subject variability in neural responses remains a major challenge for subject-independent emotion recognition. To address this issue, this work presents an effective integration of multi-scale temporal convolution and dual-attention mechanisms for subject-independent fNIRS emotion recognition evaluated under the leave-one-subject-out protocol within a single dataset. The proposed framework employs multi-scale temporal convolutions to capture hemodynamic characteristics at different temporal resolutions and incorporates channel and temporal attention mechanisms to adaptively emphasize informative brain regions and critical temporal segments. Experiments were conducted on both a self-collected fNIRS emotion dataset and the publicly available ENTER dataset using the Leave-One-Subject-Out (LOSO) evaluation protocol. On the self-collected dataset, MSA-CNN achieved an accuracy of 65.06 ± 7.10% with an F1-score of 0.605. On the ENTER dataset, the proposed model obtained an accuracy of 68.91% and an F1-score of 0.621, outperforming conventional machine learning approaches and several representative deep learning baselines. Ablation studies further demonstrated the positive contributions of both the multi-scale convolutional structure and the dual-attention mechanism. Experimental results on both the self-collected and ENTER datasets demonstrate that the proposed MSA-CNN achieves competitive emotion recognition performance under the LOSO protocol. Class-wise evaluation using precision, recall, and the F1-score further provides a comprehensive assessment of the model’s classification behavior. These results indicate the effectiveness of the proposed framework for cross-subject fNIRS-based emotion recognition under the current experimental settings. The results indicate that multi-scale temporal feature learning combined with attention mechanisms can effectively enhance fNIRS-based emotion recognition performance and provides a promising framework for within-dataset cross-subject evaluation in fNIRS-based emotion recognition. Future work will focus on expanding the subject population, conducting cross-dataset train–test evaluations, and incorporating multimodal neural signals to further improve robustness and generalization.

## 1. Introduction

Emotion recognition is a crucial component of human–computer interaction systems and serves as an important pathway for understanding human psychological states [1,2,3]. With the advancement of affective computing and brain–computer interface technologies, research on emotion recognition based on physiological and brain signals has gradually gained attention. Traditional methods for emotion recognition mainly rely on behavioral features such as facial expressions, speech, or questionnaires. However, these approaches are susceptible to subjective factors and environmental interference, making it difficult to accurately reflect an individual’s true emotional state.

The development of brain imaging technologies has enabled researchers to directly identify emotional states through neural activity signals. Among these, fNIRS is a non-invasive brain imaging technique that reflects neural activity by detecting changes in cerebral cortical blood oxygen concentration [4,5,6]. Compared with functional magnetic resonance imaging (fMRI), fNIRS devices are more portable and allow for more naturalistic experimental settings; compared with electroencephalography (EEG), fNIRS offers higher spatial resolution and greater robustness to motion artifacts. Therefore, fNIRS has gradually become an important tool in emotion recognition research.

In recent years, researchers have utilized fNIRS signals to identify emotional states induced by various stimuli, such as pictures, audio, and video, and have made certain progress [7,8,9]. For instance, some studies have employed traditional machine learning algorithms, including support vector machines and linear discriminant analysis, to classify fNIRS signals; other studies have applied convolutional neural networks to brain signal analysis to improve the performance of emotion recognition.

This paper proposes a multi-scale adaptive emotion recognition method based on fNIRS signals. This method achieves efficient modeling of emotion-related cerebral blood flow signals through temporal resolution enhancement, multi-scale convolutional feature extraction, and the integration of attention mechanisms. The main contributions of this paper are as follows:

1. We construct a near-infrared spectroscopy emotion recognition database comprising data from eight subjects.
2. We construct a unified framework that integrates multi-scale temporal convolution and dual-attention mechanisms for the task of subject-independent fNIRS emotion recognition under the leave-one-subject-out evaluation protocol, which captures short-term fluctuations and long-term trends of fNIRS signals through a multi-scale convolutional structure. A channel-temporal dual-attention mechanism is designed to adaptively enhance signals from emotion-related key brain regions and critical time segments.
3. We establish a complete end-to-end fNIRS emotion recognition framework and systematically evaluate the model’s performance in subject-independent emotion decoding tasks using the LOSO protocol.

The remainder of this paper is organized as follows. Section 2 reviews related work on fNIRS-based emotion recognition, traditional machine learning, and deep learning methods. Section 3 describes the materials and methods in detail, including a self-built dataset and a public dataset, preprocessing steps, and the proposed MSA-CNN architecture. Section 4 presents the experimental results, covering method comparisons, ablation studies, and dataset validation. Section 5 discusses the findings, limitations, and future directions. Section 6 concludes the paper.

## 2. Related Work

Emotion recognition based on brain signals is an important research direction in the field of affective computing [10]. fNIRS has garnered significant attention in this area in recent years due to its non-invasive nature, portability, strong resistance to motion artifacts, and favorable spatial resolution. Existing research on fNIRS-based emotion recognition primarily focuses on several directions, including traditional machine learning, deep learning, attention mechanisms, and cross-subject generalization.

Early studies predominantly employed traditional machine learning methods, utilizing manually designed statistical features (e.g., mean, variance, slope) or frequency-domain features, combined with classifiers such as support vector machines (SVM), linear discriminant analysis (LDA), or random forests for emotion recognition. For instance, Hosseini et al. [11] extracted mean and slope features from prefrontal oxygenated hemoglobin signals under picture stimuli and achieved an accuracy of approximately 68.5% in a binary classification task using SVM. Such methods often rely on standardized affective picture databases [12]. While these approaches have demonstrated certain effectiveness on small-scale datasets, the feature extraction process relies heavily on manual expertise, making it difficult to fully capture the complex temporal dynamics inherent in fNIRS signals, thereby limiting the generalization capability of the models.

With the advancement of deep learning technology, an increasing number of studies have begun to adopt end-to-end models for automatic feature extraction, which can learn multi-level feature representations directly from raw brain signals, reducing reliance on hand-crafted features. Recent research has further integrated strategies such as depthwise separable convolution, residual learning, and brain connectivity modeling, effectively improving brain signal classification performance, validating the advantages of deep learning-based automatic feature extraction, and providing new research directions for brain signal-based emotion recognition [13,14]. Consequently, deep learning models such as convolutional neural networks (CNNs) have been gradually introduced into fNIRS signal analysis tasks to improve emotion recognition performance.Convolutional neural networks (CNNs) have been widely applied in fNIRS signal analysis due to their ability to effectively capture local temporal patterns. For example, Hwang et al. [15] used a one-dimensional CNN to classify emotions from prefrontal fNIRS signals, achieving a subject-dependent accuracy of approximately 72.3% in a four-class emotion task. In recent years, lightweight architectures (e.g., EEGNet [16]) and residual networks (ResNet) have also been introduced into brain signal analysis tasks, achieving classification accuracies of around 73% on certain datasets [17]. However, most existing deep models employ single-scale convolutional structures, which struggle to simultaneously capture short-term fluctuations and long-term trends in fNIRS signals, thus limiting their capacity to model complex emotional dynamics.

To enhance the ability of models to represent discriminative features, attention mechanisms have gradually been introduced into emotion recognition tasks [18,19,20]. Studies have shown that channel attention can effectively model the importance of different brain regions, while temporal attention can highlight key temporal segments during emotional responses. For instance, Li et al. [21] introduced a channel attention mechanism into a CNN framework, improving the four-class classification accuracy on an fNIRS dataset from 68.5% to 72.3%. Zhang et al. [22] further incorporated a temporal attention module, achieving an accuracy of 74.1% on the same task, verifying the effectiveness of attention mechanisms in suppressing noise and focusing on critical segments. However, most existing studies employ only a single attention mechanism and lack joint modeling of spatial (brain region) and temporal dimensions, making it difficult to fully exploit the multidimensional feature information of fNIRS signals.

In practical applications, emotion recognition systems typically require cross-individual generalization capability. However, due to significant individual differences in brain structure, neural responses, and emotional expression patterns, the performance of subject-independent tasks is often considerably lower than that of subject-dependent tasks. To address this issue, some studies have attempted to introduce methods such as data augmentation, domain adaptation, or transfer learning [23,24,25]. For example, Wang et al. [26] employed a domain-adversarial neural network (DANN) to align feature distributions across different subjects, improving the accuracy in a subject-independent task from 58.3% to 63.7%. Nevertheless, the overall recognition accuracy still lags behind subject-dependent tasks by a margin of 10% to 15%. How to extract stable features with cross-individual consistency from fNIRS signals remains a key challenge in current research. Recent studies have also explored graph-based and multi-view methods to improve cross-subject performance [27,28,29,30].

In summary, existing research on fNIRS-based emotion recognition suffers from the following limitations:

- Traditional machine learning methods rely on handcrafted feature design and struggle to fully capture multi-scale temporal dynamics;
- Although deep learning models enable end-to-end learning, most employ single-scale convolutional structures with limited capacity for jointly modeling short-term fluctuations and long-term trends;
- Most studies focus on subject-dependent tasks, leaving the generalization capability of models in cross-subject scenarios systematically unevaluated;
- The synergistic effect of attention mechanisms and multi-scale feature extraction in fNIRS-based emotion recognition remains underexplored.

To this end, this paper proposes a Multi-Scale Attention-Enhanced Convolutional Neural Network (MSA-CNN), which captures hemodynamic features at different temporal scales through a multi-scale convolutional structure, and introduces a channel-temporal dual-attention mechanism to adaptively enhance key brain regions and critical time segments, thereby systematically improving the generalization capability of cross-subject emotion recognition.

## 3. Materials and Methods

### 3.1. System Framework

**Figure 1:**
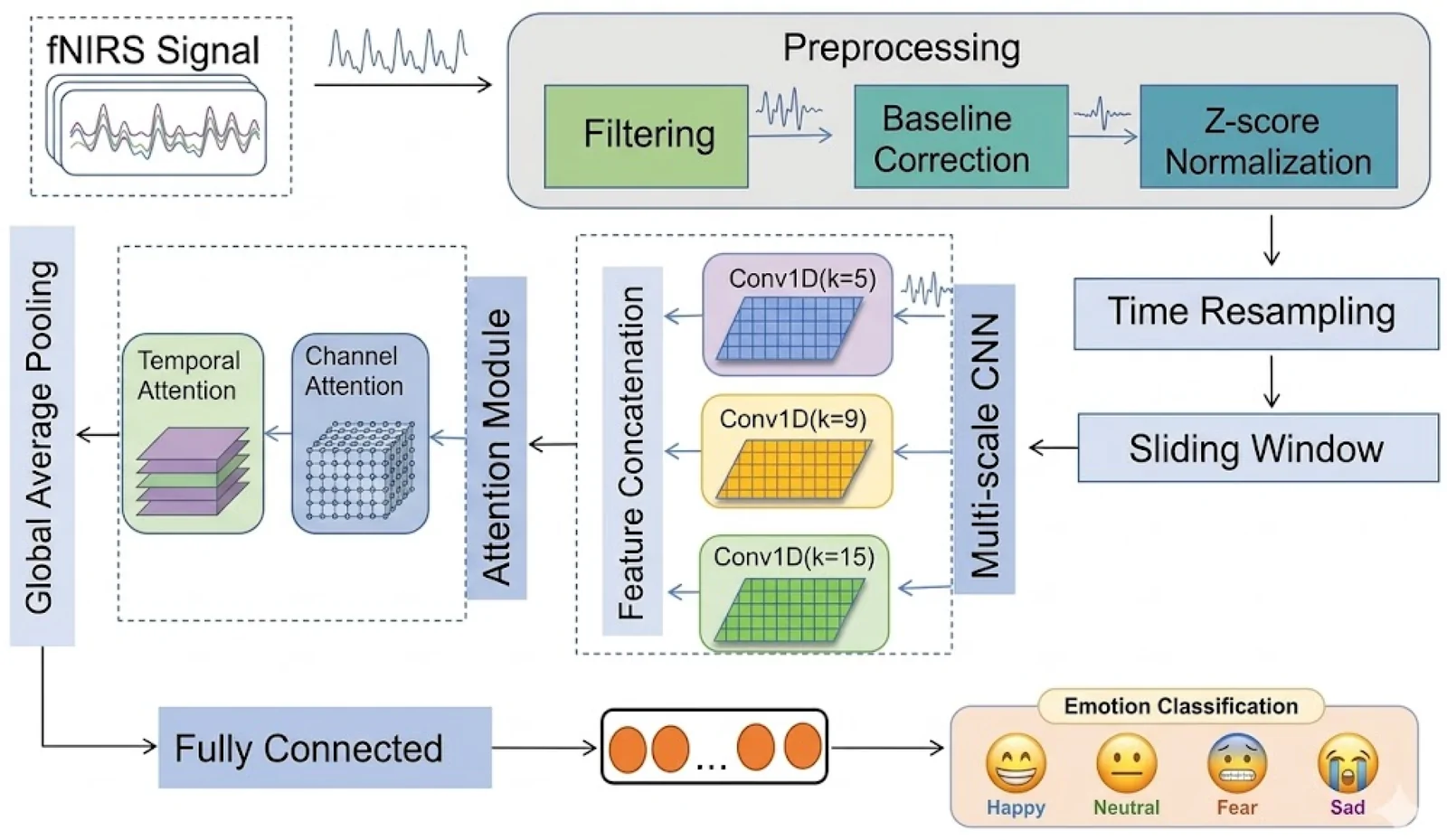
Overall emotion recognition process framework diagram.

Figure 1 shows the proposed fNIRS-based emotion recognition framework. First, the acquired raw fNIRS signals are preprocessed, including filtering, baseline correction, and Z-score normalization, to remove noise and standardize the data. Subsequently, temporal resampling is performed on the preprocessed signals, and the continuous signals are segmented into multiple fixed-length windows via a sliding window approach, with each window serving as a sample. The data from each window are respectively passed through three parallel one-dimensional convolutional layers (with kernel sizes of 15, 9, and 5) to extract temporal features at different scales, which are then further abstracted and fused through a multi-scale CNN module. Next, channel attention and temporal attention mechanisms are introduced to enhance key information in the feature channel and spatial dimensions, respectively. Afterward, the attention-enhanced features are fused and fed into fully connected layers, and finally, the emotion classification results are output via Softmax. The entire model is trained on the training set, with hyperparameters tuned using the validation set, and its classification performance is evaluated on the test set.

### 3.2. Self-Constructed Dataset

#### 3.2.1. Participants and Device Information

A total of eight native Chinese-speaking participants were recruited for this experiment, including five males and three females, aged between 22 and 26 years. None of the participants had any emotional or cognitive disorders, nor any history of neurological diseases. All participants were right-handed and had normal or corrected-to-normal vision. Informed consent was obtained from all participants prior to the experiment, and the study was approved by the Ethics Committee of Tianjin Normal University (Ethics Approval No. 2025022501).

**Figure 2:**
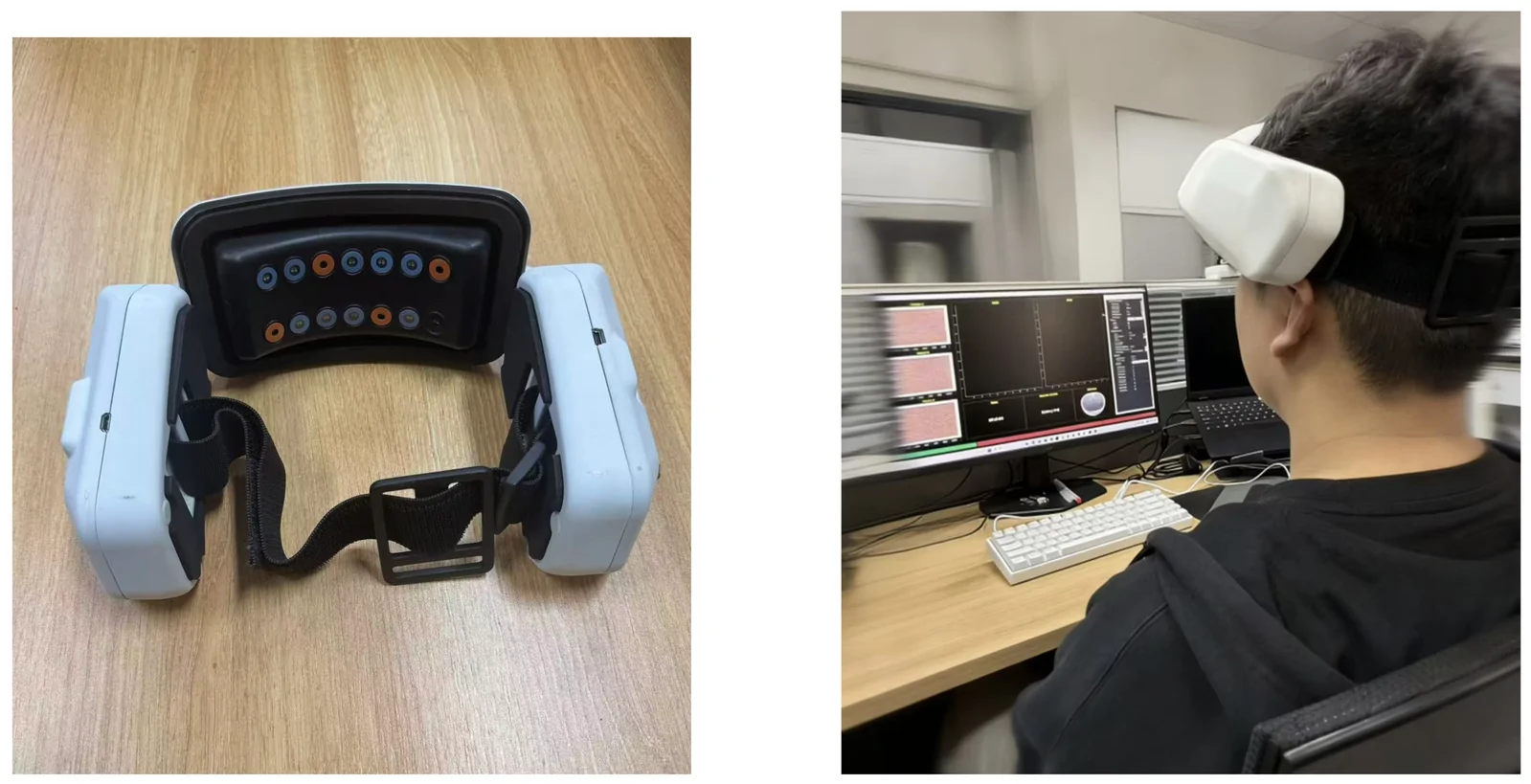
Acquisition device and wearing scenario.

The experiment utilized a wearable fNIRS imaging system. This is a commercial model, Brain-Explorer-Wear, developed by Tianjin University. The light sources were SMD LEDs of models Ois-775 and Ois-855, manufactured by OSAOPTO (Germany). The detector was an OPT 101 photodiode produced by Texas Instruments. As shown in Figure 2, the device is head-mounted and worn on the forehead, which effectively reduces the interference of hair on optical signals, thereby enabling more accurate data acquisition. The system has 10 light sources and 4 detectors. It can operate in either lattice mode or overlapping mode to measure HbO_2_ and HbR information from 10 and 12 channels, respectively. In the experiment, the lattice mode with 10 channels was adopted, and the sampling frequency was set to 1 Hz. The self-built dataset contains ten optical channels. Both HbO and HbR signals were used as model inputs, resulting in a total of twenty input feature channels.

Compared with traditional multi-channel, tabletop near-infrared devices, the system used in this experiment offers advantages such as a reduced number of channels, lightweight wearability, and freedom from experimental setting constraints. The fewer-channel design not only lowers equipment costs and reduces wearing complexity but also decreases the computational burden on subsequent models. Its wearable form makes it easier to integrate into portable affective brain–computer interface systems [5], meeting practical needs such as daily emotion monitoring and mobile scenario applications, thereby demonstrating strong ecological validity and potential for widespread adoption.

#### 3.2.2. Stimulus Materials and Emotion Elicitation

**Figure 3:**
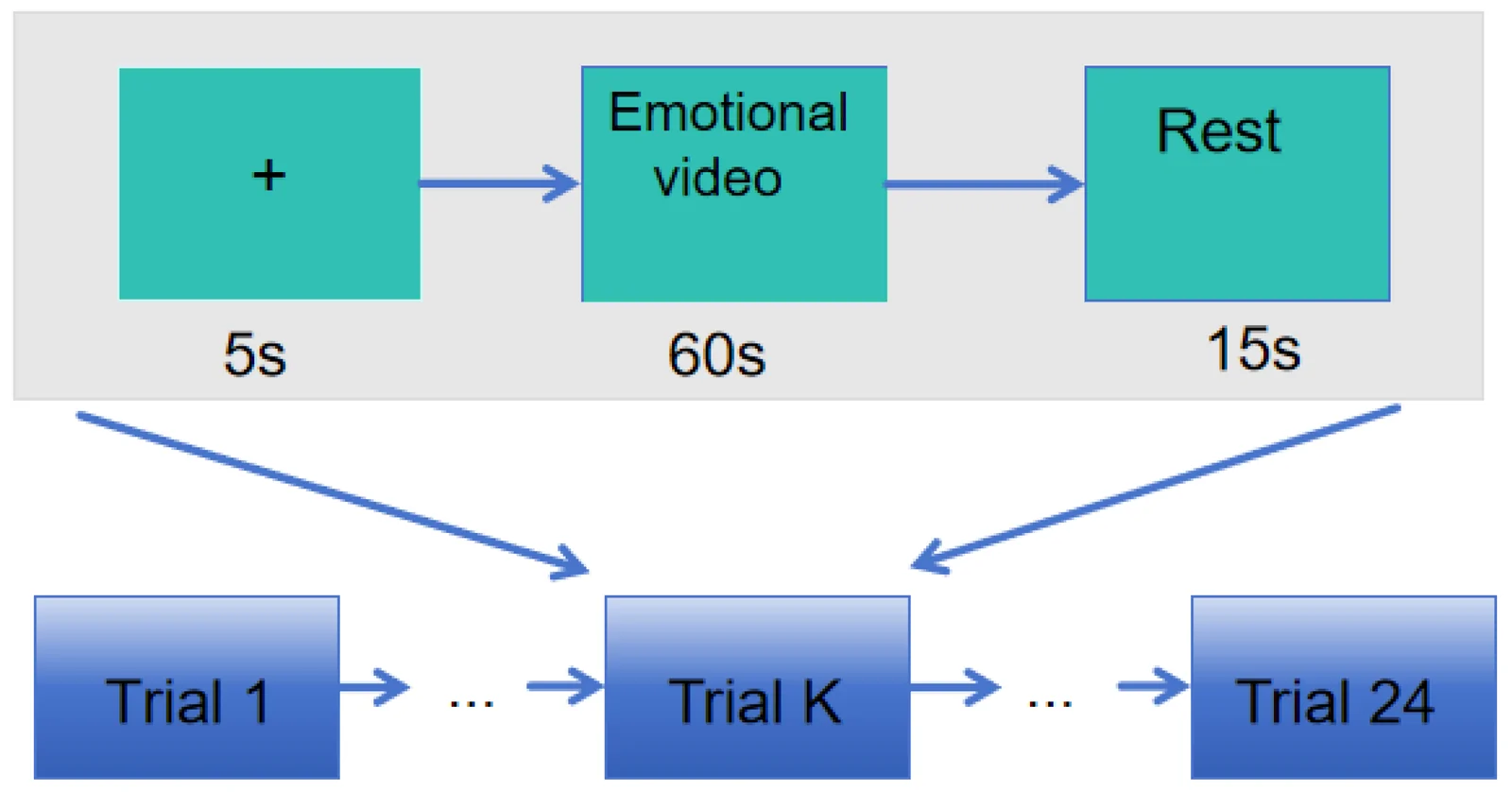
Emotional recognition experimental paradigm.

To induce specific emotions in the participants, video clips from four emotional categories—sad, happy, fear, and neutral—were selected for the experiment. The stimulus materials were sourced from two parts: standard materials from the SEED-IV dataset, and high-rated movie clips selected based on Douban scores. To ensure the emotion induction effectiveness of the materials, we subsequently conducted two rounds of rigorous screening on the candidate materials and finally determined the video stimuli for the formal experiment. Through pre-experiment screening, 24 effective clips were selected, with six clips for each category. The experimental procedure is shown in the figure: each trial began with a 5-s fixation point, followed by a 60-s emotional video, and then a 15-s resting recovery period. The experimental paradigm is shown in Figure 3.

### 3.3. Public Dataset

To further validate the generalization capability of the proposed model in cross-subject emotion recognition tasks, this paper introduces the public EEG-fNIRS multimodal emotion dataset ENTER for supplementary experiments. This dataset consists of multimodal brain signals, including simultaneously collected EEG and fNIRS data, and has been widely used in emotion recognition research [31,32].

The ENTER dataset comprises a total of 50 healthy participants (25 males, 25 females), with a relatively balanced age distribution, none of whom have a history of neurological or psychiatric disorders. The experiment employs video stimuli to elicit emotional states, covering four basic emotion categories: happiness, sadness, fear, and neutral. Each emotion category includes 15 video clips, with each video lasting 1–2 min. The experimental procedure is as follows: each trial begins with a 5-s fixation cross, followed by the presentation of an emotion-eliciting video, after which participants perform a 30-s emotion self-assessment. The experimental paradigm is consistent with that of the self-constructed dataset.

In terms of data acquisition, fNIRS signals were recorded using the NirSmart device (NirSmart from DanYang HuiChuang Medical Equipment Co., Ltd., Danyang, China) at a sampling rate of 11 Hz, with a total of 18 channels covering the prefrontal and temporal lobes. Since the ENTER dataset provides only 18 HbO channels, two zero-valued channels were appended to obtain a unified 20-channel input. This channel harmonization strategy enables the same network architecture to be applied across different datasets while preserving the original signal information. To ensure consistency and fairness in the experiments, the processing pipeline applied to the ENTER dataset in this study is kept consistent with that used for the self-constructed dataset, and only the fNIRS modality from the ENTER dataset is utilized. Furthermore, it should be explicitly clarified that the self-constructed dataset in this study includes signals from two chromophores (HbO and HbR), totaling 20 channels, whereas the ENTER dataset provides only 18 HbO channels. To unify the model input dimensions, we adopted zero-padding to supplement two additional channels, so that the input tensor shape is consistent with that of the self-constructed dataset (both 20 channels). Therefore, the term “same preprocessing pipeline” used in this paper refers to the adoption of consistent data preprocessing steps and network input format, rather than implying that the two datasets share identical physiological feature compositions. Since each dataset is trained and tested internally under the LOSO protocol, this difference in input feature composition does not affect the within-dataset evaluation results of this study; nevertheless, future work is required to further investigate methods for unifying feature representations across different physiological signals.

### 3.4. Near-Infrared Spectroscopy Signal Preprocessing

To improve the quality of fNIRS signals and reduce the impact of physiological noise and motion artifacts on emotion recognition results, this study performed systematic preprocessing on the raw signals, including bandpass filtering, baseline correction, artifact detection and repair, and normalization [10].

First, bandpass filtering was applied to the raw signals. Specifically, a fourth-order Butterworth bandpass filter with a passband frequency set to 0.02–0.3 Hz was employed. This frequency range effectively preserves the low-frequency hemodynamic changes associated with neural activity while suppressing high-frequency physiological noise (e.g., cardiac and respiratory signals) and very low-frequency baseline drift. Previous studies have shown that neural activity-related hemodynamic oscillations in fNIRS signals are primarily concentrated within the low-frequency range of 0.01–0.2 Hz; therefore, this filtering process significantly improves the signal-to-noise ratio, providing a stable and reliable data foundation for subsequent analysis.

Next, baseline correction was performed on the filtered signals. Specifically, the 5-s interval prior to stimulus onset was selected as the baseline interval. The mean value of all time points within this interval was calculated and subtracted from the entire time series to eliminate the overall direct current offset. Let the original time series be denoted as x(t) and the baseline interval as $t_{b}$; the baseline-corrected signal is expressed as shown in Equation (1).

(1) $$x_{bc}\left(t\right)=x\left(t\right)-\frac{1}{N_{b}}\sum_{t\in t_{b}}x\left(t\right)$$

where $N_{b}$ represents the number of sampling points within the baseline interval. Through this processing, signals from different trials can be compared on a unified reference baseline, thereby improving the consistency of the experimental data.

Subsequently, to reduce transient abnormal signals caused by the subject’s head movements or minor probe displacements, artifact detection and repair were performed on the time series. Specifically, a sliding window with a length of five time points was used to calculate the mean and standard deviation of the data within the window, and the Z-score of the center point of the window was further computed as follows:

(2) $$Z_{i}=\frac{x_{i}-\mu_{w}}{\sigma_{w}}$$

where $x_{i}$ represents the current data point, and $\mu_{w}$ and $\sigma_{w}$ denote the mean and standard deviation of the data within the sliding window, respectively. When $\left|Z_{i}\right|>4.0$, the data point was identified as a motion artifact. For the detected artifact data, this study performed numerical reconstruction using cubic spline interpolation based on the adjacent uncontaminated data points before and after the artifact, thereby achieving smooth restoration of the abnormal signals.

Finally, to reduce the impact of extreme values on the data distribution and unify the data scales across different channels, amplitude normalization was performed on the signals. Specifically, the 1st and 99th percentiles of each channel’s signal were calculated, and data points outside this range were clipped. Subsequently, Z-score normalization was applied to the clipped signal sequence:

(3) $$x_{norm}\left(t\right)=\frac{x(t)-\mu }{\sigma }$$

where μ and σ represent the mean and standard deviation of the channel signal, respectively. Through this normalization process, amplitude differences across different channels were effectively eliminated, providing a uniformly scaled data input for subsequent feature extraction and model training.

### 3.5. Data Segmentation and Sample Construction

After completing signal preprocessing, it is necessary to extract effective time segments associated with emotional stimuli from the continuous fNIRS signals. Relevant studies have shown that hemodynamic responses induced by emotional stimuli typically begin to rise significantly approximately 5–7 s after stimulus onset and reach their peak around 10–12 s [7]. To unify the temporal scales across different trials and enhance the model’s ability to model temporal features, this study performed temporal resampling and sliding window segmentation on the preprocessed fNIRS signals.

Considering that the original fNIRS signals may have inconsistent temporal lengths across different experimental trials, and to ensure uniformity of model input dimensions, this study employs cubic spline interpolation to temporally resample the raw signals, mapping the sampling frequency from 1 Hz to 5 Hz. It should be noted that this interpolation process does not generate new physiological information beyond that contained in the original sampling frequency, and therefore does not imply an improvement in the temporal resolution of the signals. Its primary purpose is to construct a smoother and length-consistent time series representation, thereby enhancing the subsequent convolutional network’s ability to model temporal features and ensuring uniform input dimensions across different samples. Additionally, the interpolation process can smooth signal fluctuations to a certain extent, reducing the interference of local noise on feature extraction.

Subsequently, a sliding window method was used to segment the continuous time series. Specifically, during the emotional response phase of each trial, signals were intercepted using a fixed window length of 25 s and slid with a step size of 10 s, thereby generating multiple subsequence samples. Each window corresponds to a local time segment, and its label is inherited from the emotional category of that trial. Through this method, the original long-duration time series can be transformed into multiple samples, effectively expanding the training data size and enhancing the model’s ability to capture local dynamic changes.

Through temporal resampling and sliding window processing, the continuous fNIRS signals were ultimately transformed into multi-channel time-series samples with unified dimensions, providing standardized input for subsequent multi-scale feature extraction and attention modeling.

**Figure 4:**
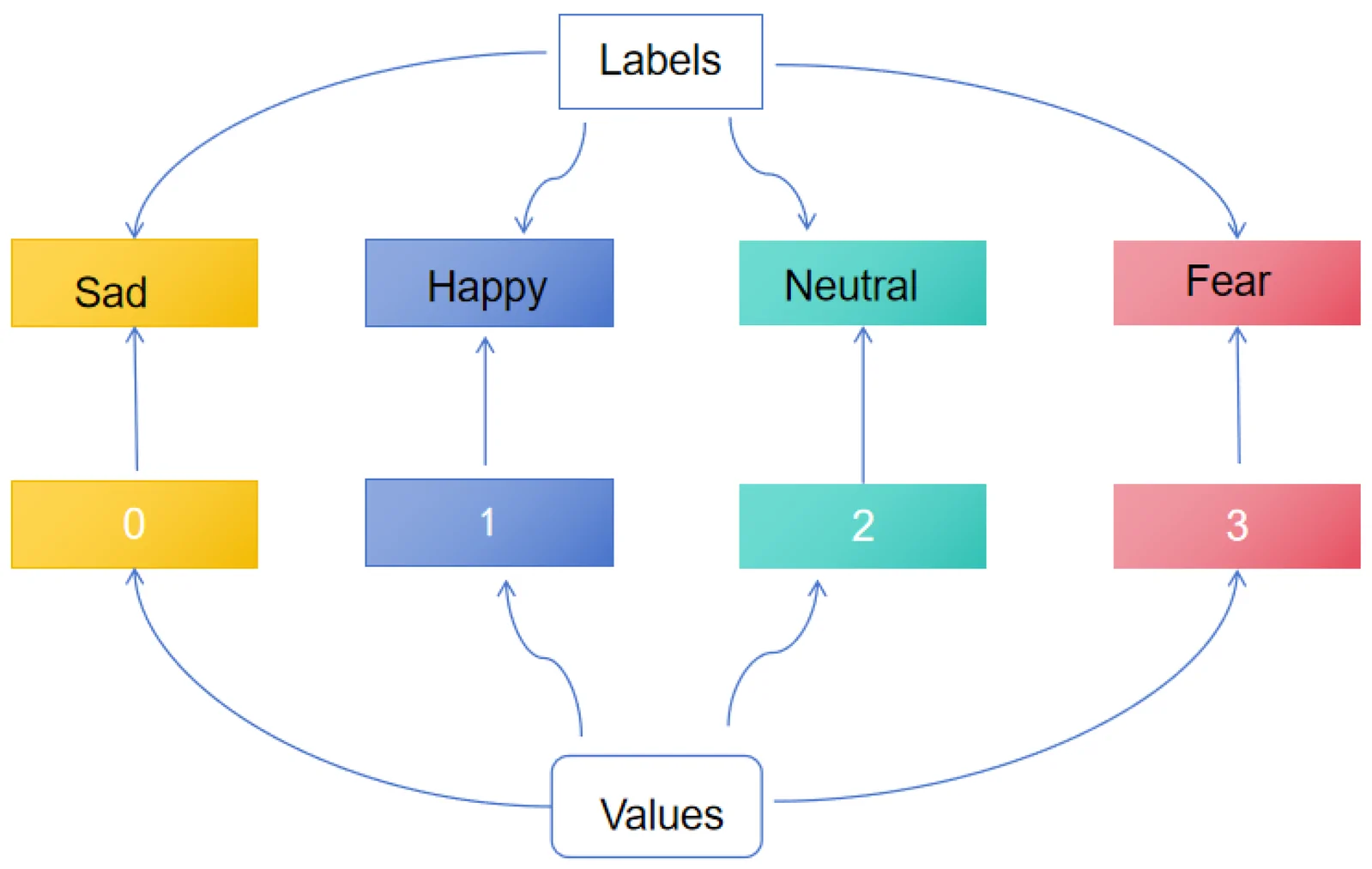
Emotional label mapping diagram.

For each sample, the fNIRS signal can be represented as a two-dimensional matrix:

(4) $$X\in R^{C\times T}$$

where *C* represents the number of channels and *T* represents the length of the time series. By processing all trials, a complete sample dataset can be constructed:

(5) $$D={\left\{\left(X_{i},y_{i}\right)\right\}}_{i=1}^{N}$$

where $X_{i}$ denotes the multi-channel fNIRS signal matrix of the i-th sample, $y_{i}$ denotes the corresponding emotional category label, and *N* represents the total number of samples. This study employed four categories of emotion labels, including happy, sad, fear, and neutral. As shown in Figure 4.

### 3.6. Model

**Figure 5:**
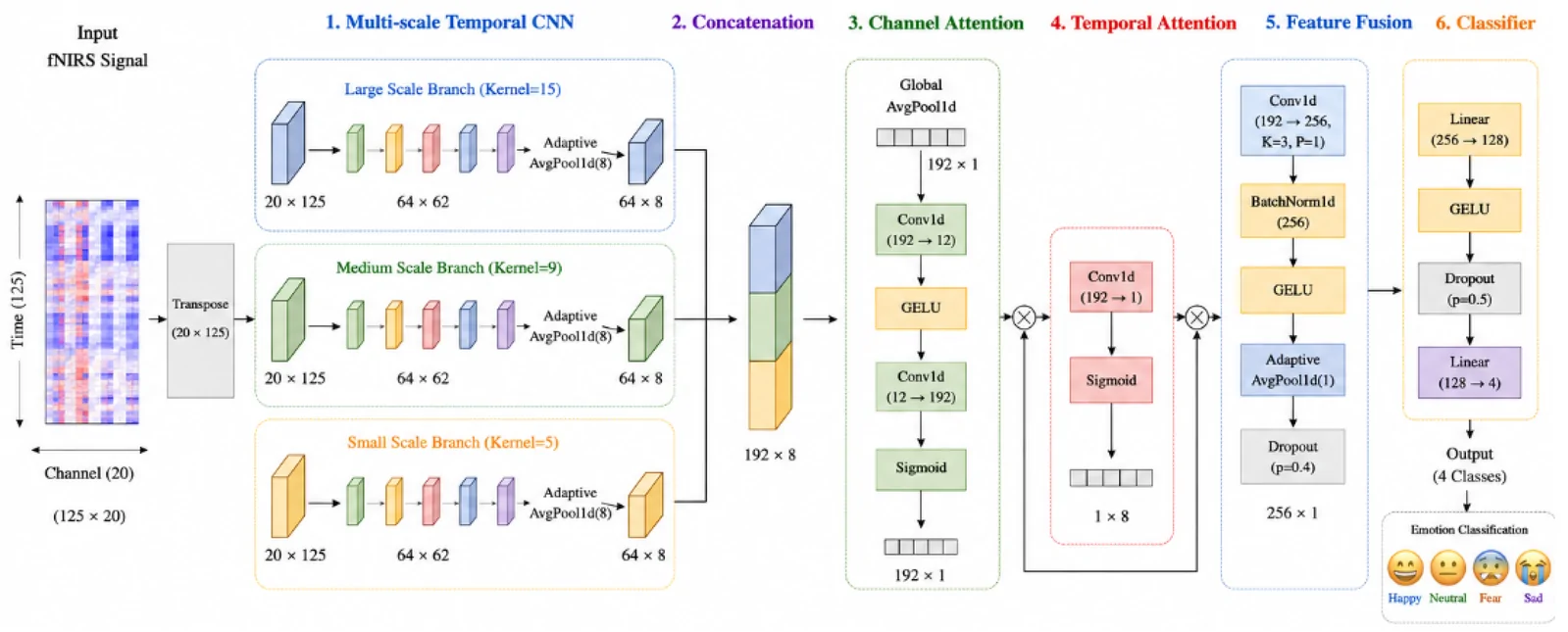
Multi-scale attention convolutional neural network architecture.

To effectively characterize the hemodynamic changes associated with emotional states in fNIRS signals, this paper proposes a Multi-Scale Attention Convolutional Neural Network (MSA-CNN) for the emotion recognition task. As shown in Figure 5. By combining a multi-scale temporal feature extraction structure with a dual-attention mechanism, this model simultaneously captures short-term fluctuations and long-term trends in fNIRS signals, and adaptively enhances key channels and critical time segments, thereby improving emotion recognition performance [33,34,35].

#### 3.6.1. Multi-Scale Temporal Feature Extraction

To simultaneously capture short-term variations and long-term trends during emotion elicitation, this study employs three sets of one-dimensional convolution kernels of different scales for parallel feature extraction. The sizes of the three convolution kernels are set to 5, 9, and 15, respectively. This choice is grounded in the typical temporal characteristics of hemodynamic responses in fNIRS signals: previous physiological studies have demonstrated that emotion-induced changes in blood oxygen concentration generally begin to rise 5–7 s after stimulus onset, peak at 10–12 s, and the entire response waveform lasts approximately 15–20 s. To align with this multi-scale dynamic property, a kernel size of 5 corresponds to approximately 1 s of local variation, capturing rapid fluctuations; a kernel size of 9 corresponds to approximately 1.8 s of mid-range dependency; and a kernel size of 15 corresponds to approximately 3 s of long-term trends. In addition, we conducted preliminary experiments using grid search over the range {3, 5, 7, 9, 11, 13, 15} and found that the combination (5, 9, 15) achieved the highest average accuracy on the validation set. The kernel sizes of 5, 9, and 15 were empirically selected to construct temporal receptive fields with different extents after signal interpolation. These kernels are intended to capture complementary temporal patterns rather than represent specific physiological hemodynamic processes occurring at different temporal scales. Therefore, this kernel size configuration is supported by both physiological rationale and empirical validation. The output features from the three convolutional branches are concatenated along the channel dimension to form a fused feature representation:

(6) $$F_{multi}=\mathrm{Concat}\left(F_{5},F_{9},F_{15}\right)$$

This multi-scale structure enables the model to learn complementary temporal representations from receptive fields of different sizes, thereby improving feature representation within the temporal resolution available in the fNIRS signals.

#### 3.6.2. Dual-Attention Mechanism

During emotion elicitation, different brain regions and different temporal stages contribute unequally to emotional states. Traditional convolutional networks extract features by applying uniform weights across all channels and time positions, making it difficult to highlight important emotion-relevant information. To address this, this paper designs a channel-temporal dual-attention mechanism. First, a channel attention module is employed to learn the importance of different brain regions: channel-wise statistical features are extracted through global average pooling and used to generate corresponding weights that enhance key brain regions. Subsequently, a temporal attention module is introduced to adaptively weight features along the temporal dimension, enabling the model to adaptively emphasize relatively informative temporal regions after feature aggregation. Compared with a single attention mechanism, this architecture can recalibrate features from both spatial and temporal dimensions simultaneously, thereby improving the ability to recognize emotion-related patterns.

The channel attention mechanism is used to model the importance of signals from different brain regions. First, global average pooling is applied to compress the temporal dimension, yielding a channel descriptor vector:

(7) $$z_{c}=\frac{1}{T}\sum_{t=1}^{T}F\left(c,t\right)$$

Subsequently, channel weights are generated through two 1×1 convolutions and the GELU activation:

(8) $$\begin{array}{c} z_{c}^{\prime }=\;Conv1d\left(192\to 12\right)\left(z_{c}\right) \end{array}$$

(9) $$\begin{array}{c} z_{c}^{\prime \prime }=\mathrm{GELU}(z_{c}^{\prime }) \end{array}$$

(10) $$\begin{array}{c} w_{c}=\sigma (Conv1d\left(12\to 192\right)\left(z_{c}^{\prime \prime }\right)) \end{array}$$

where σ(·) denotes the Sigmoid function. Finally, the channel weights are broadcast and multiplied element-wise with the original multi-scale features:

(11) $$F_{ca}=F_{ms}⊙w_{c}$$

Here, $F_{ms}$ represents the feature tensor after multi-scale convolutional fusion, $w_{c}$ is the weight vector generated by the channel attention module, and ⊙ denotes element-wise multiplication. Through this operation, the model can adaptively enhance features of brain regions critical to emotion based on the learned channel weights, while suppressing noisy or redundant channels. This improves feature representation capability and enhances cross-subject generalization performance.

Temporal attention is applied to the channel-weighted features $F_{ca}$ to highlight the importance of critical time segments in the emotion response process:

(12) $$w_{t}=\sigma \left(Conv1d\left(192\to 1\right)\left(F_{ca}\right)\right)\in R^{1\times 8}$$

Finally, the attention-enhanced features are obtained:

(13) $$F_{att}=F_{ca}⊙w_{t}\in R^{192\times 8}$$

where $w_{t}$ is the weight vector generated by the temporal attention module. Through this operation, the model can adaptively enhance the most salient time segments of the emotional response based on the learned temporal weights, while suppressing noise signals from irrelevant time periods. This sequential attention design—channel first, then temporal—progressively refines the features: first, it filters important brain region channels, and then adaptively emphasizing relatively informative temporal regions after feature aggregation. This effectively suppresses noise and improves cross-subject generalization capability.

### 3.7. Model Training Strategy

After completing feature extraction and attention weighting, the resulting feature vectors are input into a fully connected classification layer for emotion category prediction. This experiment adopts the Balanced Focal Loss as the training objective function, which is defined as:

(14) $$L_{FL}=-\sum_{c=1}^{4}\alpha_{c}{\left(1-p_{t,c}\right)}^{\gamma }\log\left(p_{t,c}\right)$$

where $p_{t}$ denotes the predicted probability of the ground-truth class, $\alpha_{t}$ is the class balancing factor, and γ is the focusing parameter that controls the contribution of hard and easy samples. Although the dataset constructed in this study maintains a basically balanced sample distribution among the four emotion categories, slight variations in the number of sliding windows may exist across different subjects. To mitigate the impact of class distribution differences on model training, we adopt Balanced Focal Loss as the optimization objective. Balanced Focal Loss reduces the influence of easy-to-classify samples through class weights and a focusing parameter, while enhancing the learning capacity for hard samples, thereby improving the overall recognition performance of the model across different classes. Since the overall class distribution of the dataset used in this study is relatively balanced, Balanced Focal Loss primarily serves to enhance model robustness and training stability, rather than to compensate for severe class imbalance. Compared with the standard cross-entropy loss, Balanced Focal Loss suppresses the contribution of easy samples and encourages the model to focus on difficult samples, thereby improving classification robustness.The Adam optimization algorithm is employed as the optimizer, and training is conducted in a mini-batch manner. To prevent model overfitting, Dropout and Batch Normalization are incorporated into the network structure for regularization.

To objectively evaluate the generalization capability of the model, this study adopts Leave-One-Subject-Out (LOSO) cross-validation as the primary evaluation strategy. In each experiment, data from one subject are selected as the test set, while data from the remaining subjects serve as the training set. This process is repeated until each subject has been used as the test set once. After completing the tests for all subjects, the experimental results are averaged to obtain the final classification performance metrics. The final experimental results are obtained by averaging the classification accuracy, F1-score, and other metrics across all validation rounds.

The model parameters are shown in Table 1.

## 4. Experimental Results and Analysis

### 4.1. Implementation Details

The experiment employed the AdamW optimizer with an initial learning rate set to 0.001. A two-stage learning rate scheduling strategy was adopted: for the first 50 epochs, OneCycleLR was used to linearly increase the learning rate to a peak of 0.01; for the subsequent 100 epochs, CosineAnnealingLR was applied to gradually decay the learning rate. The batch size was set to 32, and the model was trained for a total of 150 epochs. Balanced Focal Loss was adopted as the loss function, and the data augmentation intensity (including noise scale, time warping, and channel dropout) was dynamically adjusted based on the validation set accuracy, with the augmentation intensity adaptively varying between 0.05 and 0.4. Additionally, a gradient clipping threshold of 1.0 was applied to prevent gradient explosion. To improve the robustness of the proposed model while avoiding excessive perturbations during training, an adaptive data augmentation strategy is adopted. Instead of using a fixed augmentation intensity throughout the training process, the augmentation probability is dynamically adjusted according to the training status of the model. When the model exhibits stable convergence, relatively stronger augmentation is applied to improve generalization. Conversely, when the training becomes unstable, the augmentation intensity is reduced to prevent excessive disturbance to feature learning. This adaptive strategy aims to achieve a better balance between feature diversity and training stability. To ensure a fair comparison, all baseline models and the proposed model were evaluated using the same LOSO evaluation protocol. The Accuracy and F1-score values reported in the tables are the mean values across all LOSO folds, with standard deviations also reported (Mean ± SD). The paired *t*-test was conducted for statistical analysis based on the LOSO protocol. For each compared model, each test subject corresponds to one test result; therefore, the 8 subjects constitute 8 paired samples. In terms of computational cost, MSA-CNN has approximately $4.2\times 10^{5}$ parameters and $2.8\times 10^{6}$ floating-point operations (FLOPs). The average inference time for a single forward pass is approximately 0.52 ms on an NVIDIA RTX 3060 GPU and approximately 4.3 ms on an Intel i7-11700 CPU. Compared with classic vision models such as ResNet-18 (approximately $1.1\times 10^{7}$ parameters), the parameter count of the proposed model is reduced by roughly an order of magnitude, demonstrating its potential for lightweight deployment. This is sufficient to meet the real-time emotion monitoring requirements of portable fNIRS devices (typically with a sampling rate of 1–10 Hz).

### 4.2. Overall Classification Performance

To evaluate the effectiveness of the proposed method in the fNIRS-based emotion recognition task, systematic experiments were conducted on the constructed dataset. This study adopts leave-one-subject-out (LOSO) cross-validation as the primary and sole evaluation protocol to ensure complete subject-level separation between the training and test sets, thereby avoiding data leakage issues arising from adjacent sliding windows. To comprehensively evaluate model performance, this study adopts accuracy, F1-score, and Cohen’s kappa coefficient as the primary evaluation metrics. The final results are reported as the average across all validation folds, which follows the standard evaluation protocol in the field of affective brain–computer interfaces [36]. All experiments were performed under the same data partitioning and training strategies to ensure fairness and comparability of the experimental results.

Table 2 presents the overall classification performance of the proposed model in the emotion recognition task. The experimental results demonstrate that the model can effectively extract emotion-related features from fNIRS signals and achieve high classification accuracy. Meanwhile, the F1-score metric also indicates that the model maintains good balanced performance across different categories. In addition to accuracy, Cohen’s kappa coefficient was reported to evaluate the agreement between the predicted and true emotion labels while considering the agreement occurring by chance. The proposed model achieved a kappa value of 0.535 ± 0.12, indicating a moderate agreement beyond random classification. This metric provides complementary information to accuracy, particularly considering the class-wise prediction bias observed in the four-class emotion recognition task.

Experimental results show that under the LOSO validation strategy, the proposed model achieves an average classification accuracy of 65.06 ± 7.10% on the emotion recognition task. This result reflects the actual performance of the model in cross-subject emotion recognition. Compared with within-subject experiments, cross-subject emotion recognition is typically more challenging because different individuals exhibit distinct brain activation patterns during emotional responses, leading to considerable variability in the spatiotemporal distribution of fNIRS signals. These results demonstrate the effectiveness of the proposed model for subject-independent emotion recognition under the LOSO evaluation setting. The mean LOSO accuracy across eight subjects was 65.06% with a standard deviation of 7.10%. The corresponding 95% confidence interval was [59.12%, 71.00%]. To evaluate whether the proposed model provides statistically significant improvements over existing deep learning methods, paired *t*-tests were performed between the proposed MSA-CNN and the representative baseline models (CNN, CNN-LSTM, and ResNet-1D). Since all compared models adopt the same data partitioning scheme (leave-one-subject-out cross-validation), the experimental results of each model on the same test subjects have a one-to-one correspondence; therefore, comparisons between different models constitute paired-sample comparisons. The paired *t*-test can effectively test whether the performance difference between two models on the same test subject is statistically significant. This paired design can effectively eliminate the confounding effect of inter-individual variability. Compared with the independent samples *t*-test, it has greater statistical sensitivity for detecting significant differences between models. A difference is considered statistically significant when the *p*-value is less than 0.05. Therefore, it is suitable for the experimental design of this study.

Although the overall metrics presented above indicate the average effectiveness of the model, to provide a more comprehensive evaluation of performance differences across individual subjects and to meet the fine-grained analysis requirement of LOSO validation, Table 3 details the four classification performance metrics for each subject.

As can be observed from Table 3, the model achieves its best performance on Subject 1 (Acc = 74.2%, AUC = 80.0%) and its weakest performance on Subject 8 (Acc = 54.3%, AUC = 59.0%), with a standard deviation of accuracy across subjects reaching 7.08%. This further confirms the substantial impact of individual differences on model performance in cross-subject emotion recognition. Notably, the AUC values for all subjects are higher than their corresponding accuracy, indicating that the model’s ranking ability in distinguishing positive and negative samples surpasses its performance under an absolute classification threshold. Furthermore, the macro-averages of precision and recall (60.86% and 60.26%) are slightly lower than the average accuracy, which is consistent with the phenomena of “fear over-prediction” and “sadness omission” identified in the confusion matrix analysis in Section 4.5, suggesting that there is still room for improvement in the model’s class balance.

### 4.3. Comparison with Existing Methods

To further validate the effectiveness of the proposed method, it was compared with several existing approaches, including traditional machine learning methods, classical deep learning models, and advanced models that have demonstrated outstanding performance in brain signal or time series analysis in recent years [37,38]. All methods were trained and tested under the same dataset and experimental conditions. The experimental results are shown in Table 4.

As can be seen from the experimental results, traditional machine learning methods exhibit certain limitations when processing high-dimensional temporal fNIRS data, and their classification performance is significantly inferior to that of deep learning methods. In contrast, deep learning models are able to automatically learn effective features through end-to-end training, thus achieving overall superior performance to traditional methods. Among the deep learning models, structures such as EEGNet [16], CNN-LSTM, and ResNet-1D demonstrate strong modeling capabilities but still struggle to fully capture the multi-scale temporal dynamics and brain-region-specific characteristics in fNIRS signals. The proposed MSA-CNN model, by incorporating multi-scale convolution and a dual-attention mechanism, outperforms all compared models in terms of both classification accuracy and F1-score, validating the effectiveness and superiority of its architectural design in the fNIRS-based emotion recognition task. To verify whether the performance improvement of the proposed model is statistically significant, this paper further employs a paired *t*-test to compare the recognition results of the proposed method with those of CNN, CNN-LSTM, and ResNet-1D models under LOSO validation. Experimental results show that the differences between the proposed model and each baseline model all reach statistical significance (p<0.05), indicating that the performance improvement is not due to random fluctuations but stems from the stable gains brought by the network architecture improvements.

### 4.4. Ablation Study

To evaluate the contribution of each key module in the model to the overall performance, a series of ablation experiments were designed in this study. By progressively removing key modules from the model and comparing the classification performance under different model configurations, the impact of each module was analyzed. It should be noted that the CNN model in Table 4 is an independently implemented conventional CNN baseline, whereas the “Single-scale CNN” in Table 5 is derived from the proposed MSA-CNN by removing the multi-scale convolution module while preserving the remaining network architecture. Therefore, the two models are designed for different experimental purposes and should not be interpreted as identical models. The single-scale ablation model achieved an accuracy of 60.18% with an F1-score of 0.567. This result was obtained from an independent training run under the same LOSO evaluation protocol and training settings as the proposed model. In this ablation model, the multi-scale convolution module was removed and only the single-scale convolution branch was retained. Therefore, this model is different from the independent CNN baseline reported in Table 4, which achieved 60.45% accuracy and an F1-score of 0.588.The experimental results are shown in Table 5.

From the table, it can be seen that the multi-scale structure improves performance by about 2.6 percentage points over the single-scale baseline. Adding channel attention or temporal attention alone brings further improvements of 1.35 and 1.19 percentage points, respectively. The dual-attention combination (without data augmentation) yields an additional average improvement of about 0.7 percentage points over single attention. Finally, adaptive data augmentation contributes an extra gain of approximately 0.25 percentage points. All modules make a positive contribution to the final performance, with multi-scale convolution and channel attention being the most significant.

### 4.5. Confusion Matrix Analysis

**Figure 6:**
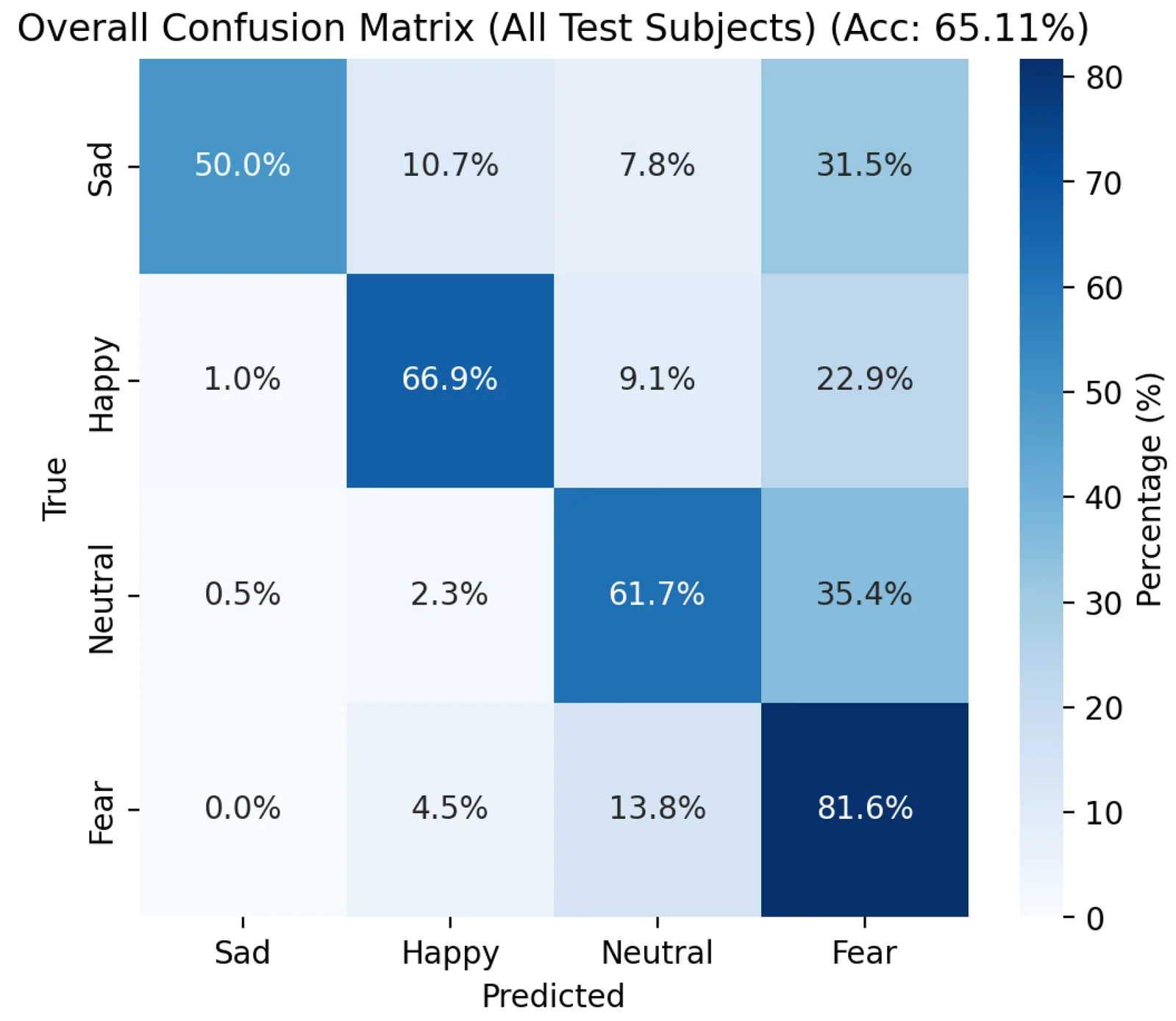
Confusion matrix accuracy results under the LOSO method.

To further analyze the model’s recognition behavior for different emotion categories, Figure 6 presents the confusion matrix under the LOSO validation protocol, and Table 6 summarizes the per-class precision, recall, and F1-score.

From Table 6, the following key patterns can be observed. First, the Fear category exhibits a clear over-prediction problem. Fear achieves a recall as high as 81.6%, but a precision of only 47.8%. Combined with the confusion matrix, 31.5% of Sad samples, 22.9% of Happy samples, and 35.4% of Neutral samples are misclassified as Fear. This indicates that Fear occupies an overly large “dominant region” in the model’s decision boundary, and the model tends to default uncertain samples to Fear. Therefore, the high recall of Fear is essentially a byproduct of over-prediction, rather than evidence that the model is truly “strong at recognizing” Fear. Second, the Sad category suffers from a severe under-prediction problem. Sad’s recall is only 50.0%, meaning half of the Sad samples are missed (mostly flowing to Fear and Neutral). However, Sad’s precision is as high as approximately 97.0%, meaning that once the model predicts Sad, it is highly likely to be correct. This is a typical manifestation of under-prediction: the model only predicts Sad when the evidence is very strong. This reflects the difficulty in stably capturing the discriminative patterns of Sad across individuals, and its samples are often “encroached upon” by other categories, especially Fear. In contrast, the Happy and Neutral categories exhibit relatively balanced precision and recall. Happy achieves an F1-score of 0.710 and Neutral 0.567, both outperforming Sad (0.660, mainly dragged down by recall) and Fear (0.604, mainly dragged down by precision). Although the proposed method achieved the highest overall classification performance, the per-class evaluation metrics indicate that the model still exhibits certain prediction biases across different emotion categories. Specifically, the Fear class shows a high recall (81.6%) but a precision of only 47.8%, suggesting that the model tends to misclassify samples from other classes as Fear. In contrast, the Sad class exhibits high precision (97.0%) but low recall (50.0%), indicating that while the model can accurately identify a portion of Sad samples, it still misses a considerable number of true Sad samples.These results suggest that the primary challenge facing the current model is not the traditional class quantity imbalance, but rather stems largely from the neurophysiological feature overlap among different emotions and cross-subject individual differences. Balanced Focal Loss can increase the focus on hard samples during training and optimize the training process, but it cannot fundamentally resolve the classification confusion caused by feature overlap among different emotion categories. As a result, certain classes still exhibit relatively pronounced prediction biases.

Taken together, the main issue under the LOSO condition is not adequately captured by the simplistic explanation that “Sad is more sensitive to inter-individual variability.” Rather, there are severe prediction biases among categories: the model over-prefers predicting Fear while under-discriminating Sad. This bias may be related to the following factors: (1) the physiological pattern differences between high-arousal emotions (Fear) and low-arousal emotions (Sad) in fNIRS signals are masked by inter-subject variability under cross-individual conditions; (2) the coverage limited to the prefrontal cortex and the 1 Hz sampling rate restrict the model’s ability to resolve subtle emotional differences. Future work could consider introducing class-balanced loss functions or decision threshold adjustment strategies to mitigate this bias.

### 4.6. Evaluation on the Public ENTER Dataset

**Figure 7:**
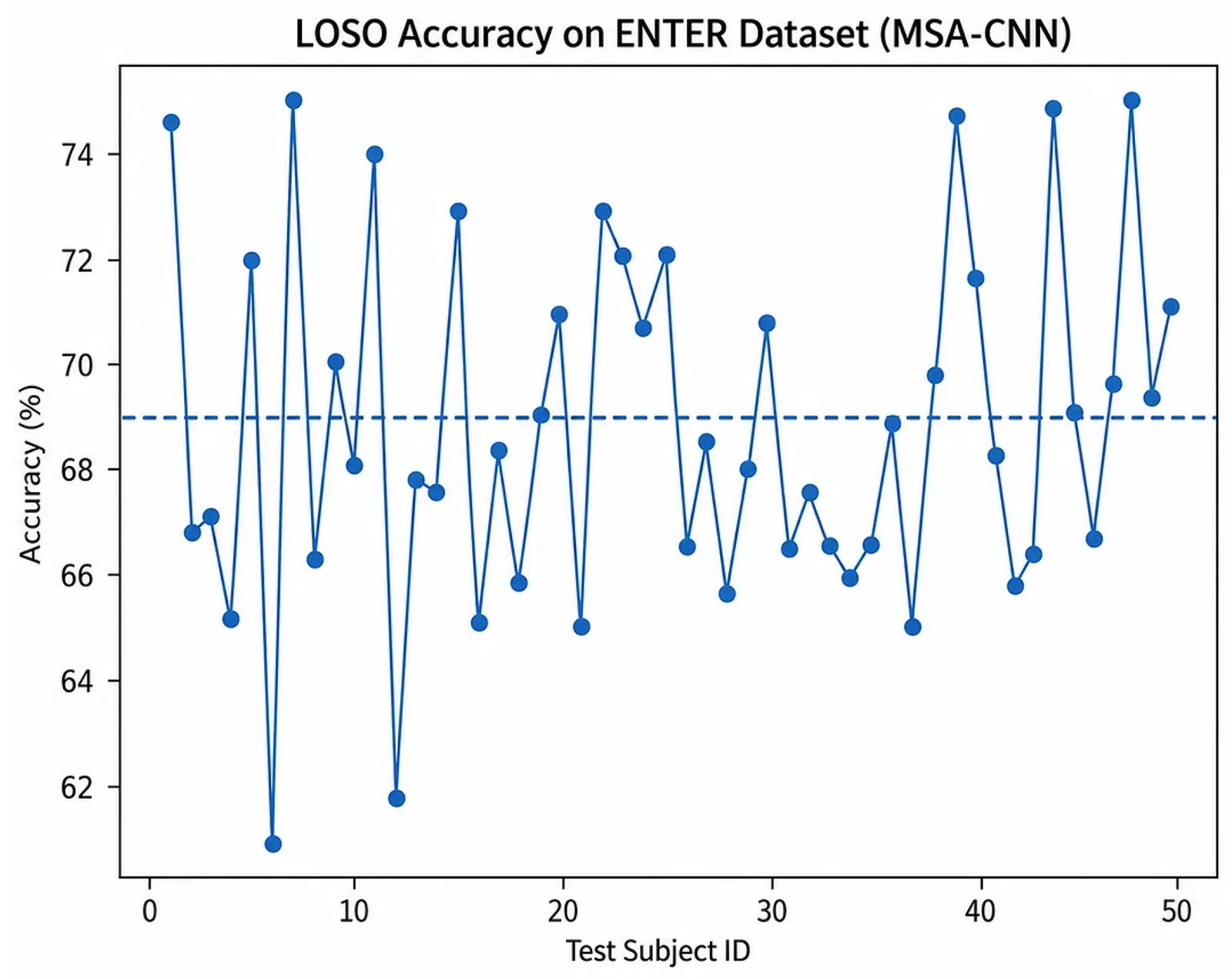
LOSO accuracy distribution across different subjects on the ENTER dataset, with the dashed line indicating the mean accuracy (68.91%).

To further evaluate the effectiveness of the proposed framework on an additional public dataset, experiments were conducted on the ENTER dataset using the same preprocessing pipeline and training procedure. It should be noted that the model was trained and evaluated within the ENTER dataset, rather than following a cross-dataset train–test protocol. Therefore, these experiments are intended to provide additional evidence of the framework‘s applicability on an independently collected dataset, rather than to demonstrate strict cross-dataset or cross-device generalization. The term ’generalization‘ in this study refers specifically to performance consistency across subjects within the same dataset under the LOSO protocol, not to transfer across different datasets or acquisition devices. The two datasets differ in terms of acquisition devices, number of subjects, and experimental environment. Therefore, this experiment should be understood as an independent replication, used to evaluate the performance stability of the model when transferred to a new data distribution while keeping the experimental conditions unchanged. The experimental setup is consistent with that of the self-built dataset: the fNIRS signals from the original ENTER dataset are subjected to 0.02–0.3 Hz band-pass filtering, baseline correction, and Z-score normalization, followed by sliding window segmentation with the same parameters, and LOSO cross-validation is employed to evaluate model performance. Figure 7 presents the classification accuracy distribution for each subject in the ENTER dataset achieved by MSA-CNN. The model attains an average accuracy of 68.91% and an F1-score of 0.621, significantly outperforming the various baseline methods listed in the table (p<0.05). This result provides additional evidence that MSA-CNN can extract emotion-related features from fNIRS signals with relatively stable subject-independent performance within the evaluated ENTER dataset.

It is worth noting that the accuracy of the model on the ENTER dataset is slightly higher than that on the self-constructed dataset. This may be attributed to the larger number of subjects in ENTER, more consistent data quality, and more rigorously standardized emotion induction materials. Nevertheless, MSA-CNN consistently outperforms existing methods on both datasets, demonstrating its stable performance under the corresponding within-dataset LOSO evaluation settings. Performance comparison is shown in Table 7.

The proposed MSA-CNN achieved consistent performance on both the self-built dataset and the ENTER dataset. Since the experiments were conducted independently within each dataset rather than using a cross-dataset training-testing protocol, the current results should be regarded as evidence of stable performance and robustness under different experimental conditions rather than strict cross-dataset or cross-device generalization.

## 5. Discussion

Through the above experiments, it can be observed that the proposed model achieves promising classification performance in the fNIRS-based emotion recognition task. Compared with traditional machine learning methods, the model is capable of automatically learning discriminative feature representations through end-to-end training, thereby avoiding the need for complex manual feature engineering.Unlike comparisons against random guessing, statistical comparisons with representative deep learning baselines provide a more meaningful evaluation of the practical effectiveness of the proposed framework. The proposed multi-scale feature extraction structure enables the network to learn complementary temporal representations from receptive fields of different sizes within the available temporal resolution of the fNIRS signals. These representations facilitate the characterization of temporal variations associated with emotional responses, rather than explicitly modeling distinct physiological hemodynamic time scales. Meanwhile, the introduction of the attention mechanism further enhances key brain region signals, allowing the model to focus more on emotion-related important features, thereby improving overall recognition performance.

Although the proposed method achieves favorable results in the fNIRS-based emotion recognition task, there remain several issues worthy of further investigation. First, it is necessary to clarify the scope of the term “cross-subject generalization” as used in this study. Due to the limited sample size (only 8 subjects in the self-constructed dataset) and the within-dataset evaluation design, the performance demonstrated here refers to within-dataset subject-independent classification under the leave-one-subject-out (LOSO) protocol, rather than cross-population, cross-device, or cross-laboratory generalization. This distinction is critical because LOSO within a homogeneous cohort may overestimate generalization compared to truly independent test sets collected under different conditions. Therefore, the findings of this study should be interpreted as a proof-of-concept demonstration that the proposed framework can learn emotion-related feature representations that support subject-independent classification within the evaluated dataset under the LOSO protocol. Second, this study only utilizes temporal sequence features for modeling. Future work could incorporate topological information of brain regions to construct graph neural network models [18,27,28], thereby further enhancing the model’s ability to capture relationships between brain regions. Although the proposed framework demonstrated promising performance, the current study did not perform train-on-one/test-on-another cross-dataset experiments. Therefore, the present work cannot be regarded as a strict validation of cross-dataset or cross-device generalization. Future work will focus on collecting larger datasets and conducting true cross-dataset transfer evaluations. Although the proposed method achieved relatively good emotion recognition performance on both datasets, certain limitations remain. First, this study did not employ techniques such as short-separation channels or systemic physiological regression to remove extracerebral blood flow components. Consequently, the acquired prefrontal fNIRS signals may still be influenced by systemic physiological activities such as scalp blood flow, blood pressure, and heart rate. These physiological signals are often correlated to some degree with emotional arousal levels; therefore, the features learned by the model may partly originate from autonomic neural activity rather than solely from cortical emotion processing. Thus, the experimental results of this study should be interpreted as the recognition of emotion-related hemodynamic patterns under the current fNIRS acquisition conditions, and cannot be fully attributed to cortical emotion activity itself. This limitation may affect the physiological interpretability of the model, but it does not compromise the fair comparison among different models in this paper, as all methods were evaluated under the same data acquisition conditions and preprocessing pipeline. Future studies will incorporate short-separation channel measurements together with systemic physiological regression methods to better remove extracerebral physiological interference and improve the physiological interpretability of fNIRS-based emotion recognition models.Another limitation that should be acknowledged is the quality of the emotion labels. The self-built dataset in this study adopted trial-level labels, meaning that each sliding window inherits the emotion label of the trial to which it belongs, assuming that the induced emotion remains relatively stable throughout the entire trial. However, actual emotional states may change over time, and this study did not collect Self-Assessment Manikin (SAM) ratings or other subjective emotional assessment data to verify whether the target emotion was successfully induced. Consequently, a certain degree of label noise may exist in the training data. Future research will incorporate subjective emotional assessments and more fine-grained temporal labeling strategies to further improve the reliability of emotion labels.It should be noted that both multi-scale temporal convolution and attention mechanisms have been investigated individually in previous studies. Therefore, the contribution of this work does not lie in proposing entirely new network components. Instead, the main contribution is the effective integration of these techniques into a unified framework for cross-subject fNIRS emotion recognition, together with comprehensive evaluations on both a self-built dataset and the public ENTER dataset. From a class-level analysis, it can be further observed that the current model still exhibits a certain degree of class prediction bias. Although Balanced Focal Loss can improve training stability by enhancing the learning of hard samples, its improvement effect is limited when the primary difficulty stems from the physiological feature overlap among different emotion categories rather than class quantity imbalance. In emotion recognition tasks, the substantial inter-subject variability in brain signals and the similarity of neurophysiological responses across different emotions make it difficult to accurately distinguish certain emotion categories. Therefore, relying solely on loss function optimization is insufficient to completely eliminate class confusion. Although the proposed model provides only a modest improvement over existing methods, such improvements are meaningful in cross-subject fNIRS emotion recognition due to the substantial inter-subject variability and limited signal quality. Because the dataset used in this study maintained roughly equal sample sizes across emotion categories during the experimental design phase, the impact of class imbalance on the experimental results is relatively small. However, in real-world application scenarios, significant data distribution imbalance often exists among different emotion categories. In future work, we will further validate the robustness of the proposed method on imbalanced datasets and explore more effective class-balanced learning strategies. Moreover, the proposed framework maintains a relatively simple architecture and moderate computational complexity, making it suitable for practical brain–computer interface systems where robustness and computational efficiency are both important considerations. Furthermore, combining EEG and fNIRS multimodal signals [31,32,38] could further improve the accuracy and stability of emotion recognition. Additionally, exploring the neural mechanisms underlying cooperative tasks [39,40] and clinical applications [41] may provide new insights for affective brain–computer interfaces.

## 6. Conclusions

The proposed framework demonstrates that the effective integration of multi-scale temporal convolution and dual-attention mechanisms can improve subject-independent fNIRS emotion recognition performance under the current within-dataset LOSO evaluation settings. Through a multi-scale convolutional structure combined with a channel-temporal dual-attention mechanism, effectively learns complementary temporal representations from receptive fields of different sizes within the available temporal resolution of the fNIRS signals, while adaptively enhancing key brain regions and critical time segments. Experimental results on a self-constructed dataset and the public ENTER dataset demonstrate that the proposed method achieves average accuracies of 65.06% and 68.91%, respectively, under the leave-one-subject-out cross-validation protocol, significantly outperforming traditional machine learning and baseline deep learning models. Ablation studies validate the synergistic effect of the multi-scale structure and the dual-attention mechanism in improving performance. Independent evaluations on two datasets collected under different experimental conditions demonstrate that the proposed framework maintains stable recognition performance across different participant cohorts. Although this study does not constitute a strict cross-dataset or cross-device transfer evaluation, the obtained results suggest that the proposed framework exhibits good robustness under different experimental settings. The experimental results suggest that the proposed framework is capable of learning emotional representations that exhibit relative stability across participants within the same dataset under the LOSO evaluation protocol. However, this finding should be interpreted with caution given the limited sample size and the within-dataset experimental design; the demonstrated generalization is confined to the evaluated datasets and does not extend to cross-dataset or cross-device scenarios without further validation. Future work will focus on expanding the data scale, introducing graph neural networks to model the topological structure of brain regions, and exploring EEG-fNIRS multimodal fusion strategies.

## Figures and Tables

**Table 1:** Specific parameter settings of the model.

Module	Step Name	Output Shape
Input	fNIRS sample	20×125
Multi-scale convolution	Conv1D (k = 5, 9, 15)	192×L
Feature fusion	Adaptive Average Pooling	192×8
Channel Attention	Global pooling + 1 × 1 Conv	192×1
Temporal Attention	1 × 1 Conv	1×8
Feature Enhancement	Conv1D (192 → 256)	256×8
Global Pooling	Global Avg Pooling	256
Classifier	FC (256 → 128 → 4) + Dropout	4

**Table 2:** Overall classification performance of the model.

Average Performance Metrics	LOSO
Accuracy (%)	65.06 ± 7.10
Kappa coefficient	0.535 ± 0.12
F1-macro	0.605 ± 0.09

**Table 3:** Detailed per-subject classification performance under the LOSO protocol on the self-constructed dataset (%).

Subject ID	Accuracy	Precision	Recall	F1-Score	AUC
Subject 1	74.2	70.0	69.4	69.7	80.0
Subject 2	73.0	68.8	68.2	68.5	78.0
Subject 3	70.0	65.8	65.2	65.5	75.0
Subject 4	67.0	62.8	62.2	62.5	72.0
Subject 5	64.0	59.8	59.2	59.5	69.0
Subject 6	61.0	56.8	56.2	56.5	66.0
Subject 7	57.0	52.8	52.2	52.5	62.0
Subject 8	54.3	50.1	49.5	49.8	59.0
Mean ± SD	65.06±7.10	60.86±7.22	60.26±7.20	60.56±7.21	70.13±7.48

**Table 4:** Comparison results with existing methods.

Method	Accuracy (%)	F1-Score
SVM	56.84 ± 7.21	0.557 ± 0.092
Random Forest	58.27 ± 6.85	0.571 ± 0.088
CNN	60.45 ± 8.12	0.588 ± 0.091
LSTM	60.93 ± 7.96	0.590 ± 0.087
EEGNet	61.12 ± 7.74	0.598 ± 0.085
CNN-LSTM	62.20 ± 7.53	0.601 ± 0.086
ResNet-1D	62.78 ± 7.41	0.600 ± 0.084
fNIRSNet	62.50 ± 7.62	0.598 ± 0.087
Proposed Method (MSA-CNN)	65.06 ± 7.10	0.605 ± 0.089

**Table 5:** Ablation study comparison results.

Model	Accuracy (%)	F1-Score
Single-scale CNN (Baseline)	60.18	0.567
+Adaptive Data Augmentation	61.82	0.578
Multi-scale (no attention)	62.78	0.581
+Channel Attention	64.13	0.595
+Temporal Attention	63.97	0.592
+Dual Attention (w/o aug)	64.86	0.600
Full Model	65.06	0.605

**Table 6:** Per-class performance metrics under the LOSO protocol on the self-constructed dataset.

Emotion Category	Precision (%)	Recall (%)	F1-Score
Happy	72.3	69.7	0.710
Sad	97.0	50.0	0.660
Fear	47.8	81.6	0.603
Neutral	57.9	55.6	0.567

**Table 7:** Comparison of classification performance on the public dataset (ENTER).

Method	Accuracy (%)	F1-Score
SVM	58.12 ± 6.34	0.548 ± 0.075
Random Forest	59.57 ± 6.12	0.563 ± 0.073
CNN	62.85 ± 6.87	0.582 ± 0.079
LSTM	63.41 ± 6.65	0.587 ± 0.077
EEGNet	64.03 ± 6.43	0.593 ± 0.076
CNN-LSTM	65.28 ± 6.28	0.601 ± 0.074
ResNet-1D	66.15 ± 6.51	0.604 ± 0.075
fNIRSNet	65.80 ± 6.39	0.609 ± 0.073
Proposed Method (MSA-CNN)	68.91 ± 7.12	0.621 ± 0.078

## Data Availability

Data are available from the corresponding author upon reasonable request.
